# Primary Abandon of Hernia Sac (PAS) Versus Complete Dissection in Laparoscopic Transabdominal Preperitoneal (TAPP) Repair of Large Inguinal and Inguinoscrotal Hernia: A Prospective Randomized Pilot Study

**DOI:** 10.7759/cureus.91844

**Published:** 2025-09-08

**Authors:** Mahmoud Aboelsaoud, Abdelazim M Elganash, Hosam M Elghadban, Mahmoud A Aziz

**Affiliations:** 1 General Surgery, Mansoura University Hospital, Mansoura, EGY; 2 General Surgery, Ibra Hospital, Ibra, OMN

**Keywords:** complete dissection, inguinal hernia, laparoscopy, primary abandon of hernia sac, transabdominal preperitoneal

## Abstract

Background: Inguinal hernia repair (IHR) is a common surgical approach globally, with laparoscopic techniques like transabdominal preperitoneal (TAPP) repair growing in popularity due to their minimally invasive nature and favorable patient outcomes. The aim of this study was to evaluate the results of the primary abandon of the hernia sac (PAS) technique in laparoscopic TAPP for large inguinal and inguinoscrotal hernias (ISH).

Methods: This prospective randomized pilot study was conducted on 53 patients aged between 16 and 70 years, diagnosed with unilateral or bilateral uncomplicated inguinoscrotal or large inguinal hernias. All participants were allocated into two groups: Group A underwent the PAS approach, while Group B underwent the complete sac dissection approach.

Results: Operative time was significantly increased in group B compared to group A (45.2±7.8 min vs. 60.6±20.1 min) (p=0.001). There were insignificant differences in postoperative seroma and hydrocele rates between the groups (p=1.0). Likewise, no significant difference was observed regarding the hernia side (p=0.893), operative complications, or other postoperative issues. Postoperative pain (POP) scores and length of hospital stay (LOS) also displayed insignificant differences between the groups.

Conclusions: The primary abandon sac technique in TAPP for large inguinal and inguinoscrotal hernias notably shortened operative time without raising rates of seroma, hydrocele, intraoperative or postoperative complications, pain, length of hospital stay, or one-year recurrence.

## Introduction

The high prevalence of hernia and its effect on quality of life (QoL) continue to represent significant healthcare concerns. Groin hernia repair is a common surgical approach. Nonetheless, the optimal surgical approach remains a subject of ongoing debate [1].

Furthermore, each approach involves some technical changes, which include surgical procedure, mesh types, mesh fixation approaches, no fixation of mesh, and attitude toward the hernia sac. These variations are designed to decrease postsurgical adverse events, pain, and recurrence; help the patient return to everyday activities rapidly; enhance QoL; and decrease postoperative discomfort and the operative adverse events [2].

The success of TAPP and totally extraperitoneal (TEP) approaches for inguinal hernias is well established. TAPP and TEP, as minimally invasive approaches (MIA), are recognized as viable treatment modalities for inguinoscrotal hernias (ISH). Laparoscopic hernioplasty has demonstrated benefits, including expedited recovery to activities of daily living (ADL) and a reduced incidence of wound infections [3].

On the other hand, the most effective procedure to repair major ISH and the optimum management of the distal sac and its adverse events remain a matter of controversy. Much research has focused on the limitations of longer operative times, especially owing to hernia sac manipulation and reduction. The dissection is conducted in a greater preperitoneal plane compared to the open approach, which involves dissecting the hernia sac from the spermatic cord without opening the preperitoneal space. Achieving complete reduction with sac transection and distal splitting can be challenging, particularly in large indirect inguinal sacs [4].

Seroma represents the most prevalent postoperative complication following laparoscopic inguinal hernia repair. A comparison between complete reduction of the hernia sac and transection revealed no significant difference in seroma frequencies (10.5% versus 10.4%). Nonetheless, the group subjected to full hernia sac reduction exhibited greater incidences of hydrocele, testicular atrophy, and cutaneous sensory alteration deficits [5].

Nevertheless, extended and meticulous laparoscopic dissection of the herniated sac elevates the probability of injury to visceral or cord structures, as well as the occurrence of seroma, hematoma, and ischemic orchitis. We hypothesize that primary abandon of the hernia sac (PAS) could potentially simplify the approach and reduce operative duration. On the other hand, residual sac tissue might augment the risk of postsurgical seroma development. To the best of our knowledge, limited information is available regarding this novel technique [6].

We aimed to assess the outcome of the PAS technique in laparoscopic TAPP for cases of large inguinal and inguinoscrotal hernias.

## Materials and methods

Sample size calculation

It was calculated by Stata Corp. 2021: Release 17. College Station, TX: Stata Corp LLC., aiming to assess the effect of the PAS technique in TAPP on operative time and postoperative complications, with respect to the prevalence of inguinal hernia reported by Jenkins and O'Dwyer [7] as 1.275%, as abdominal wall hernias had a prevalence of 1.7% and inguinal hernias represent 75% of hernias. Using confidence levels of 95%, the required minimal sample size is 20.

This prospective randomized study was conducted on 53 patients, aged between 16 and 70 years, diagnosed with unilateral or bilateral uncomplicated inguinoscrotal or large inguinal hernia. The research was carried out from October 2023 to October 2024, following approval from the Ethical Committee of Mansoura University Hospitals, Mansoura, Egypt. Informed written consent was obtained from the participants' cases.

Exclusion criteria included patients who were unfit for general anesthesia, had recurrent inguinal hernias, or presented with complicated hernias such as acute irreducibility, obstruction, or strangulation.

Randomization

A simple computer-generated software was utilized (to ensure allocation concealment and minimize selection bias, sealed opaque envelopes were employed) to divide cases into two groups: Group A underwent the PAS approach, while Group B underwent the complete sac dissection approach. Each patient underwent comprehensive history taking, physical examination, and laboratory analysis, comprising complete blood count (CBC), liver function tests (liver enzymes, serum albumin, serum bilirubin, and coagulation profile), kidney function assessment, blood glucose level, and viral markers, as well as radiological evaluations, which comprised an electrocardiogram (ECG) and abdominal ultrasound (US) to exclude the presence of intra-abdominal space-occupying lesions.

Surgical techniques

**Figure 1:**
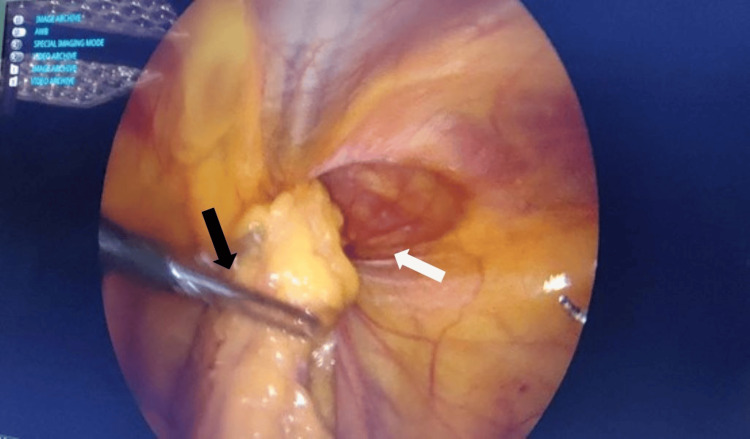
Groin exploration and content reduction The black arrow shows reduction of omentum. The white arrow shows the sac passing through the internal ring.

The procedure was conducted under general anesthesia with a single perioperative dose of intravenous Unasyn (1.5 g). Patients were positioned in the supine position with a 30-45° Trendelenburg tilt to facilitate displacement of abdominal contents. A pneumoperitoneum was established utilizing a Veress needle (closed technique), with intra-abdominal pressure maintained at 15 mmHg. The ports comprised one 11 mm supraumbilical camera port and two five mm working ports aligned along the umbilical line. A 30° laparoscope and standard laparoscopic instruments were employed. Bilateral exploration revealed the presence of direct and indirect hernias, as well as occult contralateral defects. The examination included key anatomical structures, namely, the umbilical ligaments, gonadal vessels, vas deferens, and inferior epigastric vessels, identified (Figure 1).

Primary abandon of the hernia sac approach

**Figure 2:**
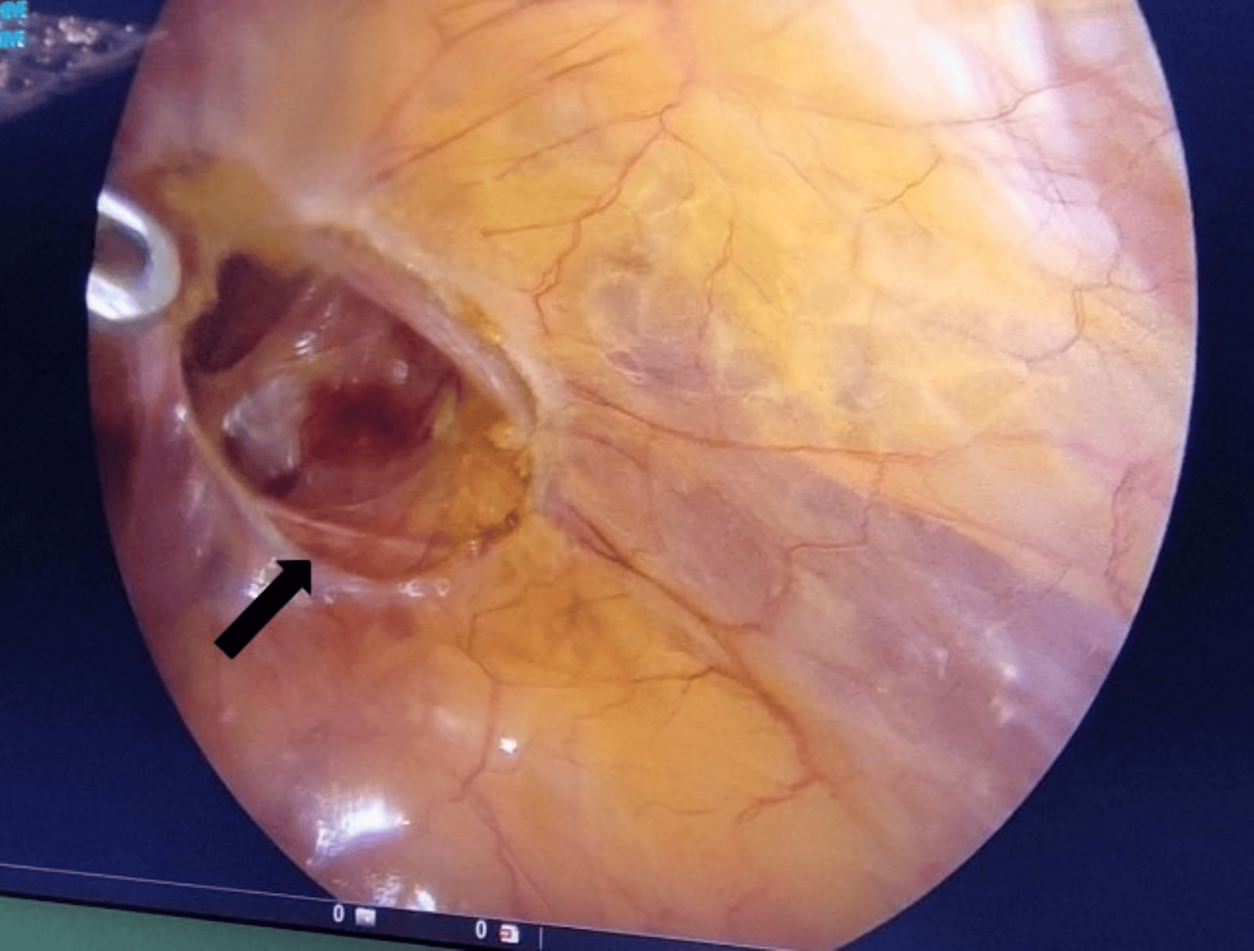
Shaping the abandoned sac Black arrow shows circular shaping of the abandoned sac

The shape of the abandon-the-sac approach is based on incomplete distal sac dissection. It involves creating a peritoneal flap that is dissected along the borders of the hernia defect, anteriorly and posteriorly, while leaving the distal hernia sac within the inguinal canal and scrotum. Following the formation of either an ellipsoid or circular shape, dissection extends medially to the medial umbilical ligament or laterally beyond the anterior superior iliac spine (ASIS). This flap provides a significant extraperitoneal surgical field quickly and easily, offering a clear view of the myopectineal orifice. By leaving the distal hernia sac within the inguinal canal in a circular shape, dissecting the cord structures located deep within the inguinal canal from the herniated peritoneum is not necessary (Figure 2).

Complete sac dissection approach

**Figure 3:**
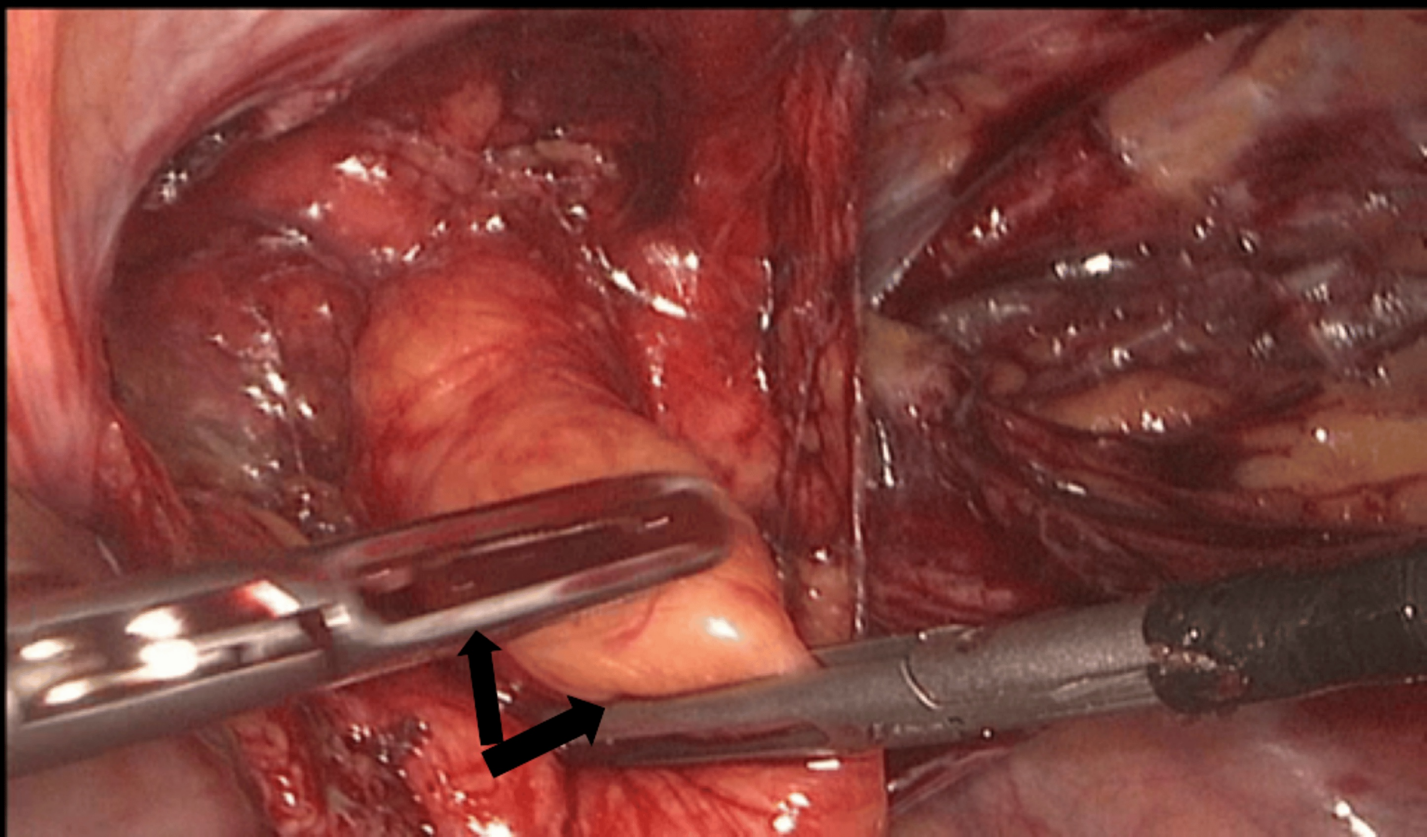
Sac dissection Black arrows show dissection of the sac.

**Figure 4:**
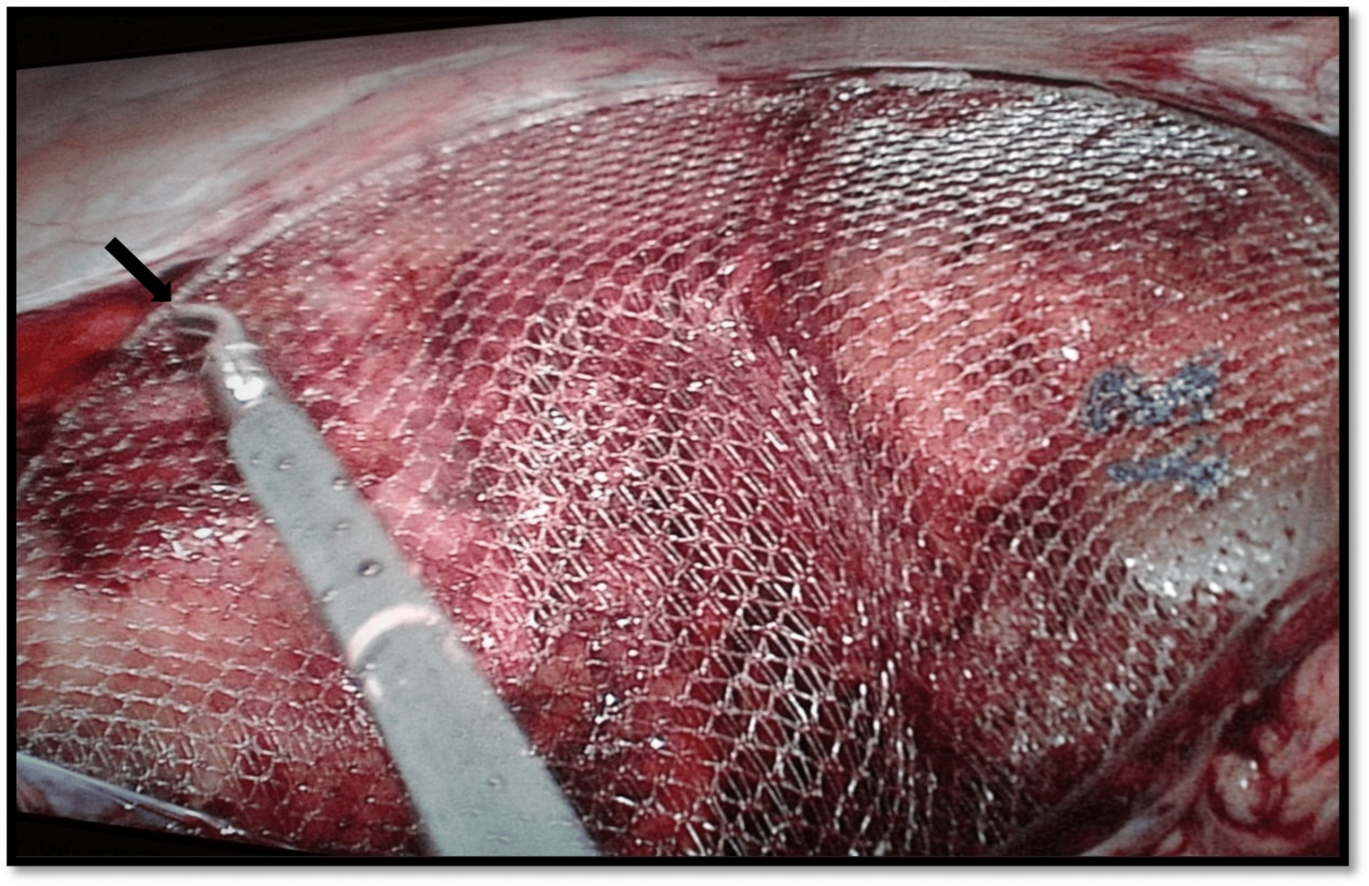
Mesh insertion The black arrow shows unfolding of the 3D mesh.

**Figure 5:**
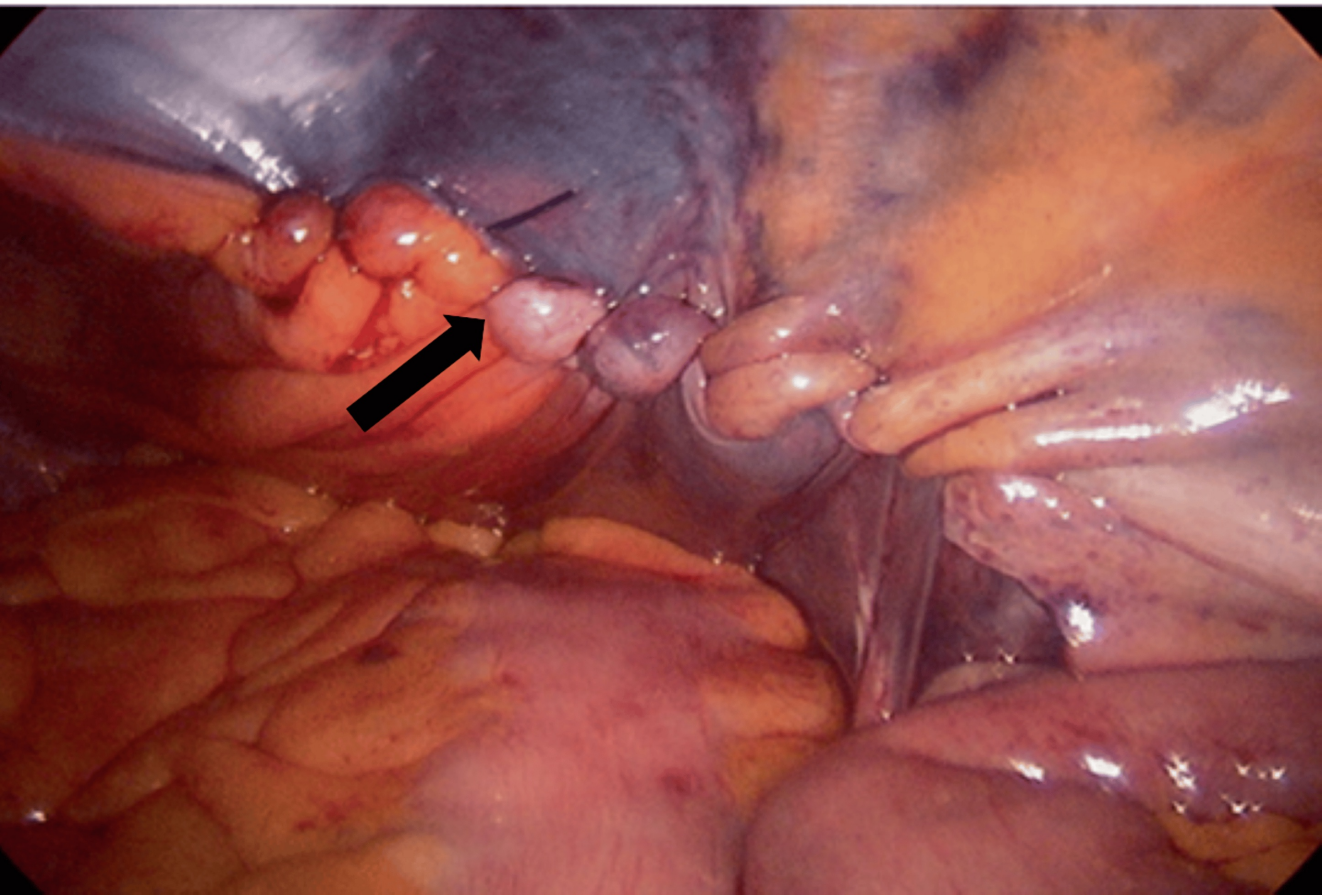
Peritoneal flap closure The black arrow shows the closed peritoneal flap.

A peritoneal flap was crafted from the ASIS to the medial umbilical ligament, thereby exposing the Bogros and Retzius spaces, Cooper’s ligament, and the ilio-pubic tract. Precautions were taken to avoid the corona mortis and critical neural structures. Hernial sac dissection from the spermatic cord components was conducted through continuous inward traction and countertraction, employing a combination of blunt and sharp dissection techniques until the musculofascial borders of the internal inguinal ring and the primary anatomical structures were adequately identified (Figure 3). A polypropylene mesh measuring 15 × 10 cm, or a 3D mesh, was tailored, inserted, and secured with absorbable tackers to cover the areas of direct, indirect, and femoral hernias (Figure 4). The peritoneal flap was closed utilizing 2-0 Vicryl sutures subsequent to pressure reduction to 8 mmHg (Figure 5). Pneumoperitoneum was then evacuated, and trocars were removed under direct visualization. The supraumbilical fascia was sutured with 0 Vicryl to mitigate the risk of port-site hernias, and the skin was closed with 2-0 Prolene sutures.

Postoperative care

Management of fluids, electrolytes, and analgesic agents (e.g., NSAIDs such as diclofenac sodium every 12 hours). Early ambulation was promoted. Scrotal support was advised to alleviate pain and reduce scrotal edema. Patients were encouraged to resume unrestricted activities at the earliest opportunity. Postoperative data collection encompassed the evaluation of POP, complications, LOS, time of returning to ADL, chronic groin pain, and recurrence.

Follow-up

Patients were followed up weekly for a month, then every three months over the following year to assess their progress.

The assessment included early postoperative complications like hematoma, ileus, abdominal distension, and bleeding, as well as late complications, which include hydrocele, wound infection, hematoma, scrotal edema, seroma, testicular atrophy, recurrence, and pain.

The duration required to restore normal daily activities was evaluated. The recurrence rate was monitored over the course of one year. Pain levels were assessed at 24 hours, one week, one month, and three months, utilizing the requirement for additional analgesics and the Visual Analog Scale (VAS), which ranges from zero (no pain) to ten (worst pain imaginable).

The main outcomes measured were the rates of seroma and hydrocele formation within one year, compared to the standard procedure. Secondary outcomes comprised operative time, intraoperative bleeding, visceral injuries, conversion to open hernioplasty, POP, LOS, time to resume normal activities, chronic groin pain, testicular atrophy, and recurrence within one year.

Statistical analysis

Statistical analysis was performed using IBM Corp. Released 2017. IBM SPSS Statistics for Windows, Version 26.0. Armonk, NY: IBM Corp. The Shapiro-Wilk test and histograms were employed to evaluate the normality of the data distribution. Quantitative parametric variables are presented as means and SD and compared between both groups with an unpaired Student's t-test. Quantitative non-parametric data are expressed as medians and IQR and analyzed using the U-test. Qualitative variables are reported as frequencies and percentages and analyzed with the chi-square test or Fisher's exact test. A p-value of less than 0.05 was considered statistically significant.

## Results

Sixty patients were evaluated for eligibility, and seven were ruled out due to not meeting the criteria. The enrolled cases were randomly divided into two groups. All patients assigned to these groups were followed and included in the statistical analysis.

There were insignificant differences between the two groups regarding age, sex, or the side of the hernia (Table 1).

**Table 1:** Comparison of age, sex, and side of hernia between studied groups Data are presented as mean ± SD or frequency (%). * Significant p-value of <0.05, t: Student’s t-test, ꭓ2=Chi-square test, FET: Fisher’s exact test.

		Group A (n=26)	Group B (n=27)	Test of sign.	p-value
Age (Years)		40.46±10.85	40.33±10.48	t=0.04	0.965
Sex	Male	26(100.0%)	25(92.6%)	FET=2.0	0.491
	Female	0(0.0%)	2(7.4%)		
Side of the hernia	Right	13(50.0%)	13(48.1%)	ꭓ^2^=0.018	0.893
	Left	13(50.0%)	14(51.9%)		

Group B was accompanied by a significant increase in the operative time compared with group A (p=0.001) (Table 2).

**Table 2:** Comparison of operative time between studied groups Data are presented as mean ± SD. A * Significant p-value of <0.05, t: Student’s t-test.

	Group A (n=26)	Group B (n=26)	Test of sign.	p-value
Operative time (minutes) Mean±SD	45.19±7.76	60.62±20.05	t=3.67	0.001*

The operative parameters indicated that a single patient was converted to open surgery due to a sliding hernia with severe adhesion, and this case was excluded from subsequent analysis. Other postoperative complications, including seroma and hydrocele, didn’t display significant differences between the groups (Table 3).

**Table 3:** Comparison of operative and postoperative complications between studied groups Data are presented as frequency (%). * Significant p-value of <0.05, ꭓ2=Chi-square test, MC: Monte Carlo test, FET: Fisher’s exact test.

		Group A (n=26)	Group B (n=26)	Test of sign.	p-value
Operative complications	No	23(88.5%)	21(80.8%)	ꭓ^2^=1.07	0.300
	Yes	3(11.5%)	5(19.2%)		
Incidence of operative complications	Visceral injury	0(0.0%)	1(3.7%)	Mc=2.27	0.686
	Peritoneal tear	2(7.7%)	3(11.1%)		
	Bleeding	1(3.7%)	1(3.7%)		
Post-operative complications	Seroma	2(7.7%)	2(7.7%)	--	1.0
	Hydrocele	1(3.8%)	2(7.7%)	FET=0.354	1.0
	Wound infection	0(0.0%)	1(3.8%)	MC=3.02	0.388
	Intraabdominal collection	0(0.0%)	1(3.8%)		
	Hematoma	0(0.0%)	1(3.8%)		

POP score, LOS, and time taken to return to ADL were insignificant between the two groups (Table 4).

**Table 4:** Comparison of postoperative pain score, length of hospital stay between studied groups, and time to resume normal daily activities Data are presented as mean ± SD or median (IQR). * Significant p-value of <0.05, ꭓ2=Chi-Square test, t: Student’s t-test.

	Group A (n=26)	Group B (n=26)	Test of sign.	p-value
Postoperative pain score	5(3-7)	5(4-8)	ꭓ^2^=1.07	0.300
Length of hospital stay (days)	1.04±0.19	1.25±0.81	t=1.34	0.184
Time to resume normal daily activities (days)	10.00 ± 2.13	10.00 ± 1.14	t = 1.54	0.453

After a one-year follow-up, there was an insignificant difference in recurrence rates between the studied groups (p=1.0) (Table 5).

**Table 5:** Comparison of recurrence rate between studied groups Data are presented as frequency (%). * Significant p-value of <0.05, FET: Fisher’s exact test.

	Group A (n=26)	Group B (n=26)	Test of sign.	p-value
Recurrence rate	0(0.0%)	1(3.8%)	FET = 1.02	p= 1.0

## Discussion

Inguinal hernia repair is a common surgical procedure worldwide, with over 20 million cases annually. Current concepts of inguinal hernia repair [8].

In our trial, seroma formation was observed in 7.7% of both the PAS and standard groups (p = 1.0), suggesting that the omission of the distal sac does not elevate the risk of fluid accumulation. These findings are in agreement with another study conducted by Li et al., which reported comparable seroma rates of 10.5% for complete reduction and 10.5% for sac transection (p = 0.94) [9]. In contrast, a prospective randomized controlled study found a higher rate of seroma in the transected group compared to the fully dissected group (18.4% versus 7.2%; p = 0.03) [10].

Hydrocele was observed in 3.8% of PAS patients in comparison to 7.7% of standard patients (p = 1.0). Li et al. [9] reported hydrocele incidences of 2.9% for transection and 5.8% for complete reduction, corroborating our observed trend toward a reduced occurrence of hydroceles associated with limited sac manipulation. Similarly, Chen et al. [11] reported a hydrocele incidence of 0.23% following single-site laparoscopic percutaneous extraperitoneal closure in pediatric patients, indicating that minimally invasive techniques could be accompanied by lower hydrocele rates. This finding disagrees with the results recorded by Lee et al., who documented a hydrocele incidence of 0.38% in patients without hernial sac removal and 0.00% in those with sac removal, suggesting that sac management strategies can influence hydrocele formation rates [12].

In our study, the PAS technique markedly decreased the average operative duration to 45.2 ± 7.8 minutes in comparison to 60.6 ± 20.1 minutes observed with the conventional TAPP approach (p = 0.001). Similarly, a study by Arrechea et al. [13] suggests that approaches minimizing peritoneal handling, like PAS, may contribute to reduced operative times.

In our study, intraoperative complications showed an insignificant difference between the two groups. This observation aligns with the findings from a study by Claus et al. [14], which noted that the differences in complication rates could be attributed to variations in surgical techniques and the extent of dissection required for each approach.

In the current study, the median VAS scores for early postoperative pain were comparable in both the PAS and standard TAPP groups, indicating that differences in sac management exert minimal influence on immediate postoperative discomfort. This finding corroborates several studies that have evaluated postoperative pain outcomes across various laparoscopic hernia repair techniques, including meta-analyses demonstrating similar pain levels whether the hernia sac is transected or fully reduced [15].

In our study, the PAS technique led to an average hospital stay of 1.04 ± 0.19 days, compared to 1.25 ± 0.81 days for the TAPP approach (p = 0.184). While this difference wasn’t significant, it indicates a tendency for shorter hospitalizations with PAS. This aligns with other laparoscopic series, where minimally invasive inguinal hernia repairs typically require only an overnight stay [16].

At one-year follow-up, recurrence was 0% in PAS compared to 3.8% in standard (p = 1.0). This result agrees with other studies. For instance, Parker et al. [17] found a 1.2% recurrence rate with PAS compared to 3.5% with complete dissection (p = 0.42).

The limitations of the current study comprise a relatively small sample size and its single-center design. Therefore, we recommend considering the PAS technique as a viable alternative to complete sac dissection in laparoscopic TAPP repair for inguinal hernias. Its adoption may result in reduced operative times and potentially fewer intraoperative complications, without elevating the risk of postoperative issues or hernia recurrence. Surgeons are encouraged to consider integrating the PAS technique into their practice, especially in cases where complete sac dissection presents technical difficulties. Further research involving larger sample sizes and extended follow-up periods is suggested to prove or disprove the current results and evaluate long-term outcomes.

## Conclusions

This RCT demonstrates that using the Primary Abandon Sac technique in TAPP for large inguinal and inguinoscrotal hernias significantly shortens operative time. Importantly, it does so without increasing risks of seroma, hydrocele, intraoperative or postsurgical complications, pain, LOS, or hernia recurrence within one year. These findings, confirmed by multiple randomized controlled trials (RCTs), cohort studies, and a meta-analysis, endorse PAS as a safe and effective alternative to complete sac dissection. Further multicenter research and investigations are necessary to better understand its role in hernia repair paradigms.
